# Triggering receptor expressed on myeloid cells-1 deletion in mice attenuates high-fat diet-induced obesity

**DOI:** 10.3389/fendo.2022.983827

**Published:** 2023-01-09

**Authors:** Benjamin Brustolin, Nina Touly, Marine Maillefer, Lola Parisot, Elisa Di Pillo, Marc Derive, Sébastien Gibot

**Affiliations:** ^1^ Inserm Unité Mixte de Recherche (UMR) S1116, Faculté de Médecine de Nancy, Université de Lorraine, Vandœuvre-lès-Nancy, France; ^2^ INOTREM, University of Lorraine, Nancy, France; ^3^ Service de Médecine Intensive Réanimation, Hôpital Central, Nancy, France

**Keywords:** Obesity, TREM-1, adipocyte, inflammation, insulin resistance

## Abstract

**Introduction:**

The low-grade inflammatory state present in obesity leads to the development and perpetuation of comorbidities associated with obesity. Our laboratory has been working for several years on an amplification loop of the inflammatory response mediated by TREM-1 (Triggering Receptor of Expressed on Myeloid Cells-1). It is implicated in many acute (septic shock) and chronic (IBD) inflammatory diseases. Previously, TREM-1 has been shown to be overexpressed in adipose and liver tissue in obese and diabetic patients, but its impact has never been characterized in these pathologies.

**Methods:**

Our hypothesis is that TREM-1 plays a major role in the generation and perpetuation of inflammation during obesity and its associated complication (Insulin resistance and cardiac dysfunction). We assessed TREM-1 protein expression by western blot and immunofluorescence in omental and subcutaneous (pre-)adipocyte. Moreover, we submitted mice to a high-fat diet and investigated the effects of the genetic *Trem1* deletion (*trem1* KO mice).

**Results:**

We showed, for the first time, that TREM-1 is expressed and is functional in subcutaneous and omental (pre-)adipocytes. In the mouse model of high-fat diet-induced obesity, we found that *Trem1* suppression limited weight gain, insulin resistance and inflammation in white adipose tissue and liver.

**Discussion/conclusion:**

Our results reveal the *trem1* KO model can be viewed as a preventive model and that TREM-1 seems to play an important role in the development of obesity and its associated complication. It could therefore be a new therapeutic target in this context.

## 1 Introduction

Obesity is a complex and heterogeneous disease in which the interplay between adipocytes and adipose tissue macrophages or other surrounding immune cells (CD4, CD8, NKT, mastocytes…) drives the development of a chronic inflammatory state (1). This inflammation is considered to be responsible for insulin resistance that causes or exacerbates obesity-associated complications such as type 2 diabetes mellitus, atherosclerosis, hypertension, liver, kidney, or heart dysfunction (2). In this context, numerous agents have been tested in pre-clinical models of obesity, from curcumin to inhibitors of Interleukin-1 beta (3, 4). However, none of these successfully applied to humans. Indeed, as of today, only 6 anti-obesity medications have been approved by the FDA, all of them promoting weight loss through appetite suppression or decreased fat absorption (5).

The Triggering Receptor Expressed on Myeloid cells-1 (TREM-1) is an immune receptor mostly expressed at the membrane of neutrophils, mature monocytes, and macrophages (6). Following activation of Toll-Like Receptors (TLRs), the expression of TREM-1 is up-regulated and dimerization occurs, allowing the binding of its still unknown ligand that triggers the association of TREM-1 with the adaptor protein DAP-12, leading to intracellular signaling (6–8). The engagement of TREM-1 yields to an amplification of the inflammatory response (6, 9). The role of TREM-1 in mounting a dysregulated and deleterious inflammation has been proven in acute diseases or syndromes such as septic shock (9), myocardial infarction (10), or stroke (11), but also in chronic disorders: inflammatory bowel diseases, atherosclerosis, arthritis, lupus, and cancer (12–16).

Vasquez et al. showed that serum concentrations of the soluble form of TREM-1 (sTREM-1), which is known to be a marker of the TREM-1 pathway activation (17), were elevated in non-obese young healthy adults with cardiometabolic risk factors (abdominal obesity, fasting hyperglycemia, hypertriglyceridemia, high blood pressure) (18).

To get more insights into the role of TREM-1 in obesity, we submitted mice to a high-fat diet and investigated the effects of the genetic *Trem1* deletion on obesity development and its consequences.

## 2 Material and methods

### 2.1 Cells maintenance, differentiation, and stimulation

Cryopreserved, subcutaneous and omental preadipocytes from male donors with normal body mass index (22.5 +/- 1.5 kg/m²) were purchased from Zen-Bio Inc (Durham, USA). Cells were maintained at 5% CO_2_ in preadipocyte DMEM/Ham’s F-12 medium from Zen-Bio with 3.15 g/L D-glucose (17.5 mmol/L), HEPES, Fetal Bovine Serum (FBS), Penicillin, Streptomycin, Amphotericin B. Cells were seeded into T75 flasks and expanded once before differentiating using accutase (Sigma-Aldrich, France). The medium was replaced every 2 days.

For differentiation, the preadipocyte medium was replaced with DMEM-Ham’s F-12 medium supplemented with HEPES, FBS, biotin, pantothenate, human Insulin, dexamethasone, IBMX, PPARγ agonist, penicillin, streptomycin, and amphotericin B during 7 days. Cells were then fed by removing some of the media and replacing it with fresh adipocyte DMEM/Ham’s F-12 medium containing HEPES, FBS, biotin, pantothenate, human insulin, dexamethasone, penicillin, streptomycin and amphotericin B (Zen-Bio, USA). 2 weeks after the initiation of differentiation, cells appear with large lipid droplets and are considered mature adipocytes.

Each *in vitro* experiment was conducted using preadipocytes from at least three individual donors. Human preadipocytes or adipocytes were incubated under resting conditions or with LPS from Escherichia coli 0127:B8 at 100 ng/ml (Sigma-Aldrich, France) with or without TREM-1 agonists at 5 µg/ml (MAB1278, Biotechne, R&D Systems, USA) or IgG control (Biotechne, R&D Systems, USA) at 2, 4, 6 and 24h. Cells supernatants and protein lysates were recovered to perform cytokine and protein assays respectively.

### 2.2 Confocal microscopy

Cells were seeded and differentiated or not on LabTek chambers (Thermo Fisher Scientific, USA). Cells were washed using PBS and tween 20 (0.05%) and fixed with paraformaldehyde (4%) for 20 min and permeabilized with Triton 0.1% for 30 min before incubation with 1/200 anti-TREM-1-AF488 and 1/200 anti-DAP12-AF555 antibodies (Bioss, USA) at 4°C overnight. Nuclei were stained with TO-PRO-3 at 1 µg/ml (Invitrogen, USA) for 1h at 37°C. After washing, coverslips were mounted using a Vectashield solution (Vector Laboratories, USA). Confocal images were obtained using a Leica SP5 confocal laser-scanning microscope system (Leica, Germany) fitted with appropriated filter sets and acquired in sequential scan mode.

### 2.3 TREM-1 protein analysis

Pre-adipocytes and adipocytes proteins were extracted using RIPA extraction buffer containing (1% NP-40, 1% sodium deoxycholate, 0.1% SDS, 0.15 M NaCl, 0.01 M sodium phosphate pH 7.2, 2mM EDTA, 50mM sodium fluoride, 0.2 mM orthovanate sodium, 100 U/ml protease inhibitor) (Abcam, U.K). Mouse liver proteins were extracted using PhosphoSafe buffer (Novagen, Merck Biosciences, Nottingham, U.K). Total protein concentration was measured by Pierce BCA Protein Assay Kit (Thermo Fisher Scientific, USA) and used for protein normalization. Lysates were then analyzed by western blot. Electrophoresis was performed with 10 µg of proteins per well using a polyacrylamide Criterion XT Bis-Tris Gel, 4-12%, and XTMES buffer (Bio-Rad, USA). Migration was carried out at 170V, 3A, and 300W for 30 minutes. Then, proteins were transferred on a 0.2 µm nitrocellulose membrane (Transblot Turbo Transfer Pack, Bio-Rad, USA) with a Transblot Turbo system set to 2.5A, 25V for 7 minutes (Bio-Rad, USA). Coomassie blue (Thermo Fisher Scientific, USA) and ponceau (Sigma-Aldrich, France) stains were performed on the membrane and the gel respectively to control the transfer. TREM-1 (Bio-Rad, USA) antibody with its corresponding secondary antibody conjugated to horseradish peroxidase (Bio-Rad, USA) and Super-Signal West Femto Substrate (Thermo Fisher Scientific, USA) were used for protein detection. GAPDH antibody (Cell Signalling Technology, USA) was used for normalization on the same membranes after stripping for 20 minutes in stripping buffer (1% tween, 1.5% glycine, and 0.1% SDS, pH 2.2). For mouse liver analysis mouse p-ERK/ERK, p-mTOR/mTOR, p-GSK3αβ/GSK3αβ, Glut1, Glut2, p-AKT/AKT, IRα, IRβ, and GAPDH with their corresponding secondary antibodies were used. The acquisition was performed with LAS-4000 imager (Fujifilm, Japan) and Multi-Gauge software (LifeScience Fujifilm, Japan).

### 2.4 Cytokines measurements

Supernatants from stimulated cells were recovered after 2, 4, 6, and 24h LPS (100 ng/ml) stimulation. MCP-1, IL-6, and IL-8 were assessed by multiplex assay using the ELLA microfluidic system (Ella Automated Immunoassay Systems, R&D Systems, U.K) according to the supplier’s recommendation.

### 2.5 Animals

The homozygous knock-out animals were produced at Charles River (Saint Germain sur l’Arbresle, France), AALAC-accredited mouse breeder. Mating duos or trios are established for maintaining and producing animals. Animals remain undisturbed during pregnancy; newborns are genotyped by PCR screening on tail biopsies taken at 2 weeks’ of age. Colony maintenance is done in compliance with the 3-R guidelines. Animal health and welfare are verified during bedding changes. All experimentations involving animals have been carried out according to the French and European institutional legislation concerning the care and use of laboratory animals and have been approved by the French ethics committee (Number 23273) for animal experimentation. 20 six-week-old male *Trem1* knockout (KO) and 20 C57Bl/6 wild-type littermate (WT) mice were used. Mice were housed in ventilated racks placed in an EOPS-Type environment maintained at a temperature of 21 +/- 1°C, an humidity of 55% +/- 10%, and exposed to a cycle of 12 hours of light for 12 hours of darkness. The animal had free access to water and food. Four groups were constituted: 10 littermate C57Bl/6 wild-type mice with a standard diet (LFD Low Fat Diet), 10 littermate C57Bl/6 wild-type mice with a high- fat diet (HFD), 10 *Trem1* ko mice with a standard diet, and 10 *Trem1* ko mice with a high- fat diet. High-fat diet was composed of 60.6% of the calories from lipids, 17% from proteins, and 22.5% from carbohydrates (Safe-Lab, France). 10 grams of food per day and per mouse were distributed in the feeders and at the bottom of the cages to facilitate access to food. The food was weighed and extended 3 times per week in to monitor calorie consumption. Mice were weighed weekly to monitor weight gain. The diet started after two weeks of acclimatization, for 12 weeks.

### 2.6 Oral glucose tolerance test

The procedure used to perform glucose tolerance tests is based on the work of Andrikopoulos et al. (19) taking into account the influence of the glucose administration route, dosage, fasting time, and the influence of anesthesia. The oral glucose tolerance tests were performed following 10 weeks of diet feeding. Mice were fasted for 6 hours before the test and transferred to a procedure room. Blood was obtained by removing the distal 2 mm of the tail and was assessed for baseline glucose levels using the Accutrend^®^ Plus glucometer (Roche, Switzerland). Mice then received 2g/kg body weight of a 100 mg/ml glucose solution in sterile water. 20 µl of blood was collected at each time and glucose concentration was measured at 15, 30, 60, and 120 min after the administration of glucose by oral gavage. Plasma samples were collected and stored at -80°C for measuring insulin levels. Plasma insulin concentrations were determined in the same mice by a commercially available mouse insulin ELISA kit (Merck, Germany).

### 2.7 Real-time quantitative PCR

Frozen liver, subcutaneous, mesenteric, and perigonadal white adipose tissue were cut into small pieces, kept frozen, and ground in a homogenizer (FastPrep-24, MP biomedicals SARL, France) using ceramic balls 1.4 mm in diameter in the presence of Trizol reagent (Thermo Fisher Scientific, USA) to extract total RNA. RNA purification was performed using RNAeasy Plus Mini Kit (Qiagen, Netherlands) according to the manufacturer’s instructions and quantified with MicroDrop (Thermo Fisher Scientific, USA) to determine RNA purity and concentration. Samples were diluted to the same concentration before being reverse transcribed using the iScript cDNA synthesis kit (Bio-Rad, USA) and quantified by quantitative polymerase chain reaction (PCR) using Qiagen probes (Quantitect Primers) for murine *Tnfa*, *Il6*, and *Ccl2*. Murine *Gapdh*, *ActinB*, *Rna18S* probes served as housekeeping genes.

### 2.8 Statistical analyses

Unless otherwise stated, data were normally distributed and are presented as mean +/- SEM. The normality of the data distribution was determined by the Shapiro-Wilk test. Between-group differences were tested using Student’s t-test or ANOVA or with Mann-Whitney or Kruskal Wallis tests when appropriate. Statistical analysis was performed using Prism Version 8.0.1 software (GraphPad, USA). P-value < 0.05 was deemed significant.

## 3 Results

### 3.1 TREM-1 is expressed by (pre-) adipocytes and is functional

Although TREM-1 is known to be mostly expressed by myeloid cells, it has been found on the surface of some epithelial and endothelial cells.

**Figure 1:**
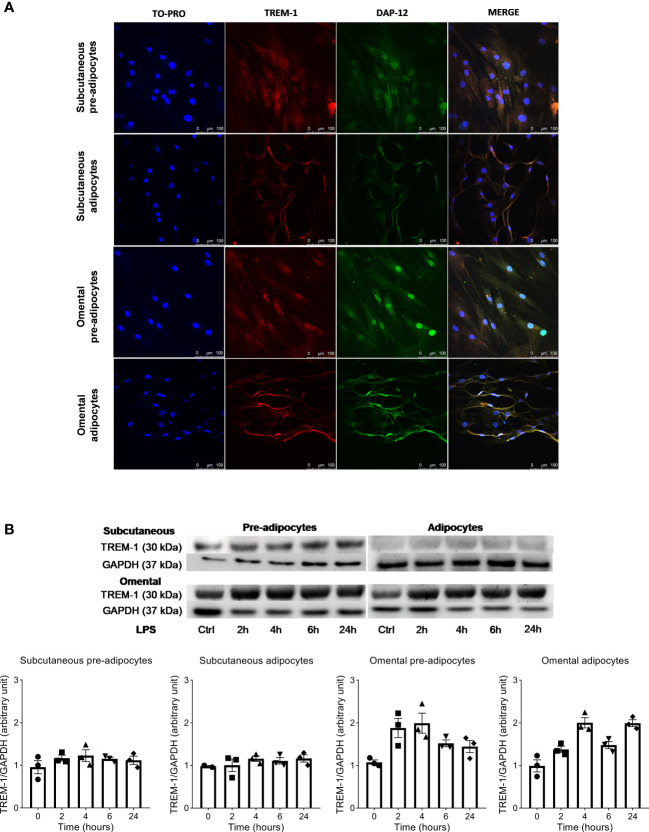
Triggering Receptor Expressed on Myeloid cells-1 (TREM-1) is expressed by human subcutaneous and omental pre-adipocyte and adipocytes. **(A)** Confocal fluorescent images of human subcutaneous (top panels) and omental (bottom panels) pre-adipocytes and adipocytes. Cells were stained with anti-human TREM-1 (red), DAP-12 (green), and TO-PRO3 (blue) mAbs, Scale bars: 100 µm. **(B)** Western blot of lysates of human subcutaneous and omental pre-adipocytes and adipocytes treated for 2, 4, 6, and 24 hours with LPS (100 ng/ml). Images are representatives from 3 different experiments each performed on three different donors.

Confocal microscopy analysis revealed the presence of TREM-1 along with its adaptive protein DAP-12 in unstimulated subcutaneous and omental pre-adipocytes and adipocytes (**Figure 1A**). We confirmed this finding by Western blot (**Figure 1B**). Moreover, we observed that TREM-1 was more expressed by omental than subcutaneous cells and that lipopolysaccharide (LPS) increased its expression.

**Figure 2:**
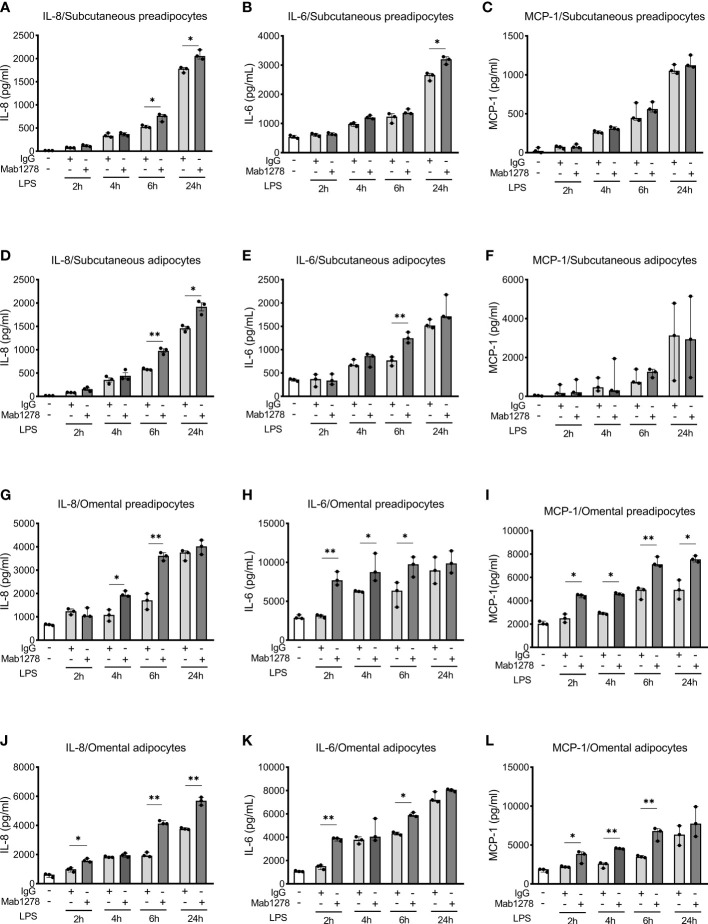
Activation of TREM-1 increases LPS-induced inflammation in human subcutaneous and omental pre-adipocyte and adipocytes. Automated multiplex ELISA quantification of IL-8, IL-6, and MCP-1 in cell supernatants from subcutaneous pre-adipocytes **(A–C)**, adipocytes **(D–F)**, and omental pre-adipocytes **(G–I)**, adipocytes **(J–L)**. Cells were incubated under resting conditions or stimulated with LPS (100 ng/ml) with or without TREM-1 agonist (MAB1278 at 5µg/ml) or control immunoglobulin G (IgG) for indicated times. Data are representative of 3 independent experiments each performed on three different donors. The significance between the IgG and MAB1278 conditions was determined using the Mann-Whitney test *p < 0.05, **p < 0.01. Data are presented as median and 95% confidence interval.

To decipher whether TREM-1 was functional, we stimulated cells with LPS along with a TREM-1 agonist antibody (MAB1278, Bio-Techne, clone 193015) or a control IgG and measured IL-8, IL-6, and MCP-1 concentrations in the supernatants (**Figure 2**). LPS stimulation time-dependently increased the concentrations of these proteins. Co-stimulation with MAB1278 potentiated this elevation, more in omental than subcutaneous (pre-) adipocytes following the higher expression of TREM-1 on the former.

Thus, TREM-1 is expressed by (pre-)adipocytes and is functional.

### 3.2 *Trem1* deletion limits weight gain in mice fed with a high-fat diet

**Figure 3:**
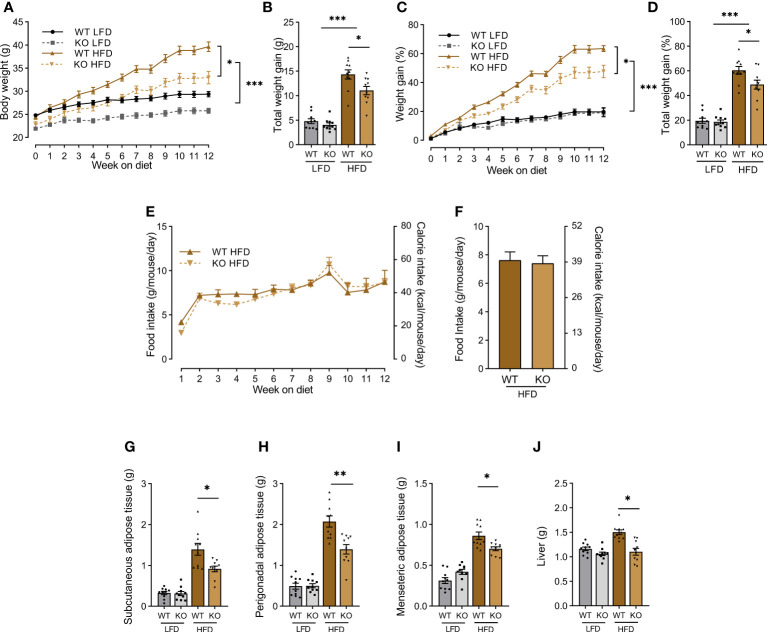
*Trem1* deletion limits weight gain in HFD mice. **(A)** Body weights of Wild-Type (WT) or TREM-1 Knock-Out (KO) mice fed with control Low Fat Diet (LFD) or High Fat Diet (HFD) during 12 weeks. **(B)** Total body weight gain (g), **(C)** body weight gain (%), **(D)** total body weight gain (%), **(E)** food and calorie intake over 12 weeks, **(F)** mean of food and calorie intake over 12 weeks in WT and KO mice fed with LFD or HFD (n=10 mice per groups One-way ANOVA analysis). **(G)** subcutaneous, **(H)** perigonadal, **(I)** mesenteric adipose tissue, and **(J)** liver weight after 12 weeks of feeding of WT and KO LFD and HFD mice. (n=10 mice per group Student’s *t*-test analysis). Data are presented as mean ± SEM (*p < 0.05, **p < 0.01, ***p < 0.001).

TREM-1 KO mice and wild-type (WT) littermates were fed with a low (LFD) or high fat diet (HFD) and followed up for 12 weeks. As expected, weight gain was modest in LFD mice, whatever the genotype (**Figures 3A–D**). By contrast, HFD animals became obese but weight gain was lower in TREM-1 KO than in WT mice (47% +/- 4,11% vs. 60% +/- 3,73%, p<0.05) (**Figures 3A–D**). Of note, food and calorie intake were similar in TREM-1 KO and WT animals all along the experiment (**Figures 3E, F**). Subcutaneous, perigonadal, and mesenteric adipose tissue, as well as liver, were less heavy in KO than in WT mice (**Figures 3G–J**).

*Trem-1* genetic ablation reduces weight gain, and adipose tissue and liver enlargement during HFD.

### 3.3 Inflammation is reduced in TREM-1 KO mice

**Figure 4:**
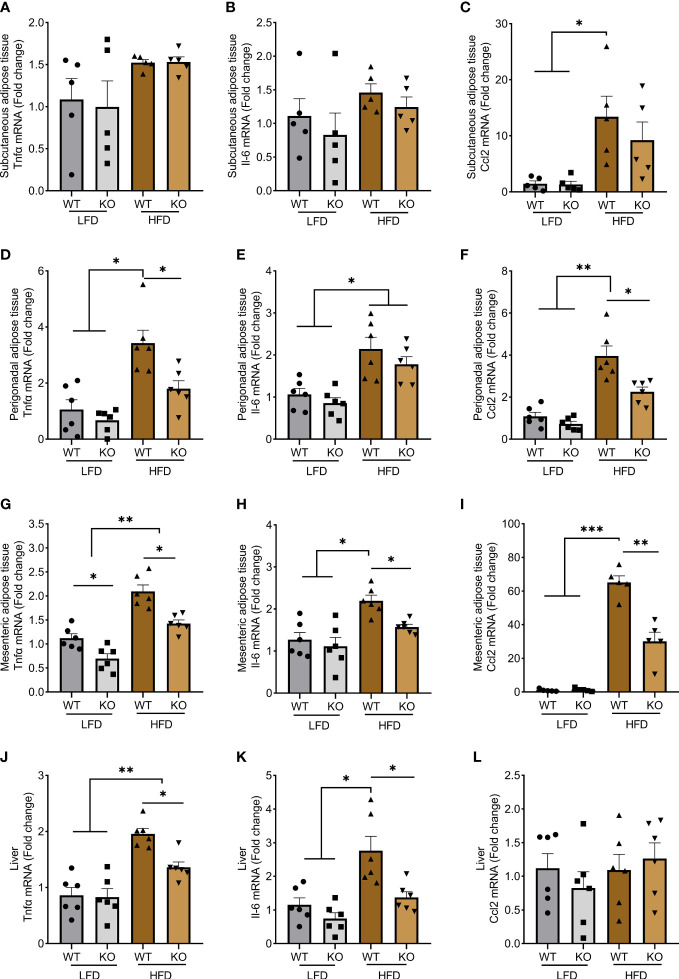
*Trem1* deletion decreases inflammation in obese mice. Quantification of *Tnfα*, *Il6*, and *Ccl2* mRNA expression by RT-qPCR in **(A–C)** subcutaneous, **(D–F)** perigonadal, **(G–I)** mesenteric adipose tissue, and **(J–L)** liver from WT or TREM-1 KO mice fed with control LFD or HFD. Data are presented as mean ± SEM, n=5-6 mice per group (Kruskal Wallis test *p < 0.05, **p < 0.01, ***p < 0.001).

To investigate the inflammatory response induced by HFD, we quantified *Tnfa*, *Il6*, and *Ccl2* mRNA by qRT-PCR in adipose and liver tissues (**Figure 4**). Expression of these genes remained at a low level after an LFD, with no difference between genotypes except for a mild reduction of *Tnfa* in mesenteric adipose tissue from KO mice (**Figure 4G**). By contrast, *Tnfa*, *Il6*, and *Ccl2* mRNA increased in most tissues following HFD. With the noticeable exception of subcutaneous adipose tissue (**Figures 4A–C**), *Trem1* deletion was associated with a decrease in these cytokines expression as compared to WT littermates (**Figures 4D–L**), but importantly, inflammation was not totally abrogated.

Adipose tissue macrophages have an important role in generating or perpetuating inflammation. We, therefore, quantified macrophage infiltration in adipose tissues by qRT-PCR. HFD increased the macrophage population as assessed by higher *Adgre1* gene expression (encoding for the macrophage marker F4/80) in subcutaneous, perigonadal, and mesenteric adipose tissues (**Supplementary Figure 1**). In these tissues, *Itagx* (CD11c) may be considered a marker of type 1 (M1) and *Mrc1* (CD206) a marker for type 2 (M2) macrophages. We observed that HFD mostly induced an increase of M1 macrophages with few effects on M2 (except in perigonadal tissue). This was reduced in TREM-1 KO mice.

Therefore, *Trem1* deletion dampens HFD-induced inflammation in several adipose tissues and the liver.

### 3.4 Trem1 deletion protects against insulin resistance

**Figure 5:**
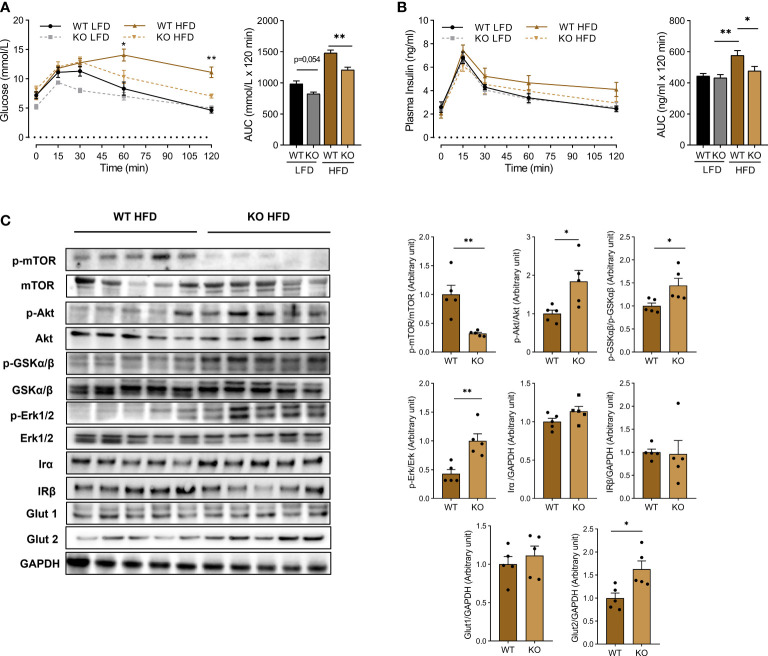
*Trem1* deletion dampens HFD-induced insulin resistance liver. **(A)** Blood glucose and **(B)** insulin were measured during an oral glucose tolerance test on WT or TREM-1 KO mice fed with control LFD or HFD after 10 weeks of diet. Mice were fasted for 6 hours before the test. (AUC: Area Under the Curve). Data were presented mean ± SEM, n=5 mice per group. Brown-Forsythe and Welch ANOVA were used for Glucose and insulin measurement to compare WT HFD vs KO HFD (*p < 0.05, **p < 0.01). **(C)** Western blot of liver lysates and their densitometric quantification. Data were presented as mean ± SEM, n=5 mice per group (Mann-Whitney test *p < 0.05, **p < 0.01).

One of the major consequences of obesity is insulin resistance which may lead to type 2 diabetes and its related complications. We performed an oral glucose tolerance test in LFD and HFD mice. As expected, HFD animals displayed higher blood glucose concentrations than LFD (**Figure 5A**). However, hyperglycemia was reduced and shortened in TREM-1 KO mice as compared to WT littermates. In parallel, hyperinsulinemia was more pronounced in WT than in TREM-1 KO HFD mice, witnessing the presence of insulin resistance (**Figure 5B**).

To get insights into the mechanisms of insulin resistance, we performed a western blot analysis of crucial proteins involved in this phenomenon (**Figure 5C**). Glucose transporters GLUT1 and insulin receptor (IR-α and IR-β) expression did not differ regarding the genotype. By contrast, activation of mTOR, known to down-regulate insulin signaling (20), was reduced in TREM-1 KO. On another side, glucose transporter GLUT2 and AKT, GSK3, and ERK phosphorylation was increased in TREM-1 KO animals: these pathways have been shown to promote cell survival and growth through their action on insulin-stimulated glucose uptake, glycogen, and protein synthesis (21). These data show that *Trem1* deletion prevents HFD-induced insulin resistance.

As part of the deleterious consequences of obesity, heart dysfunction may occur. We thus evaluated cardiac function at the end of the 12 weeks of HFD. Echocardiography revealed a decrease in left ventricular fractional shortening in HFD WT but not in TREM-1 KO mice (**Supplementary Figure 2**).

## 4 Discussion

Here we show that TREM-1 is expressed and functional in (pre-) adipocytes and that its genetic ablation confers favorable effects in HFD mice including reduction of weight gain, inflammation, and insulin resistance.

Although TREM-1 was initially considered to be only expressed by myeloid cells (neutrophils, monocytes/macrophages), it has been observed in many other cell types including epithelial or endothelial cells (22, 23). Whatever its cellular localization, TREM-1 activation leads to an amplification of the inflammatory response triggered by the previous engagement of TLRs. The deleterious role of TREM-1 over-activation is established in acute diseases such as septic shock (9) or myocardial infarction (10), as well as during chronic disorders such as atherosclerosis (13).

In 187 young, healthy, non-obese adults, Vasquez et al. showed that those with several cardiometabolic risk factors (hypertension, hypertriglyceridemia, low HDL, or abdominal obesity) had higher serum concentrations of sTREM-1 (18), a biomarker of the TREM-1 pathway activation (24), and suggested that the follow-up of this marker may alert on the development of a future metabolic syndrome.

In a small population (n=22) of obese patients, Subramanian et al. observed the presence of TREM-1 in tissue biopsies (liver, omental, and subcutaneous tissues), as well as elevated plasma sTREM-1 concentrations as compared to non-obese control subjects (25). Interestingly, TREM-1 expression was higher in diabetic obese as compared to non-diabetic obese patients, suggesting an association between insulin resistance and TREM-1 activation.

As adipose tissue is made of many different cell types (adipocytes, myeloid cells, stromal cells…), we first confirmed the presence of TREM-1 in pre-, and adipocytes and observed that its expression seems to be higher in omental than in subcutaneous adipose cells. Moreover, TREM-1 expression was upregulated following LPS stimulation in omental (pre-)adipocytes. In line with its role in amplifying the inflammatory response, we observed that TREM-1 activation increased the production of some cytokines and chemokines by adipose cells, especially the omental ones.

Having demonstrated the expression and functionality of TREM-1 in adipose cells, we moved to the classical diet-induced obesity model in mice. Although the difference is not statistically significant, the body weight of TREM-1 KO mice is slightly lower than wild mice of the same age. To study the evolution of weight gain, we expressed the results in percentages. We observed that the genetic ablation of *Trem1* dampened HFD-induced weight gain, inflammation, and insulin resistance, hallmarks of obesity. Although the precise mechanisms by which TREM-1 inhibition confers protection remain to be elucidated, we hypothesized that the reduction of the HFD-induced inflammation may be the *primum movens* preventing the development of insulin resistance and thus breaking the vicious circle (HFD induces inflammation that leads to insulin resistance that alters energy use and fat deposition and perpetuates inflammation). Indeed, Li et al. (26) showed that oxidized low-density lipoprotein (oxLDL), whose production is increased during HFD, can activate TREM-1 that in turn contributes to atherogenesis and foam cell formation by enhancing proinflammatory cytokine production. The link between oxLDL and TREM-1-induced inflammation has recently been confirmed by Wang et al. (27).

However, as we used TREM-1 KO animals, we are left with the question of the cell type(s) involved in this protective genotype: is it the deletion of *Trem1* on adipocytes, myeloid cells, or both that confers beneficial effects? Further studies are needed using adipocyte-specific *Trem1* deletion, or irradiated TREM-1 KO mice reconstituted with bone marrow from WT littermates.

The TREM-1 KO model can be viewed as a ‘preventive’ model, and then another important question arises: is the modulation of TREM-1 able to reverse an established phenotype? To address this we could use the TREM-1 inhibitory peptide Nangibotide (under clinical study in septic shock patients) but this would require repeated injection as its half-life is very short (28). The administration of an antagonist antibody would be more practical but it does not exist yet.

Several questions remain unanswered. We were able to determine the mechanisms involved in weight gain differences. Food intake monitoring indicated it was not due to fluctuation in calorie intake and that *Trem1* ablation didn’t modify leptin secretion. TREM-1 could have affected food assimilation by influencing the intestinal microbiota or energy expenditure through variation in the population of beige adipocytes. Moreover, it is necessary to determine whether *Trem1* deletion could provide long-term protection for mice from the development of obesity-related comorbidities. Finally, we could not determine whether the reduction in insulin resistance was due to weight differences or to a more direct protective effect of the *Trem1* deletion. Anyway, TREM-1 activation seems to have a role in diet-induced obesity in mice by reducing weight gain, liver inflammation and protecting against associated comorbidities such as insulin resistance and cardiac dysfunction. Its pharmacological modulation deserves further investigation with the aim to break the vicious circle of insulin resistance and inflammation.

## Data availability statement

The original contributions presented in the study are included in the article/**Supplementary Material**. Further inquiries can be directed to the corresponding author.

## Ethics statement

The animal study was reviewed and approved by French ethics committee (Number 23273).

## Author contributions

BB and SG conceived the study. BB, NT, MM, LP, and ED performed experiments. MD reviewed the data. BB and SG wrote the first draft of the manuscript. All authors read and approved the final version of the manuscript. All authors contributed to the article and approved the submitted version.
