# Supplementary figures and images for: Triggering receptor expressed on myeloid cells-1 deletion in mice attenuates high-fat diet-induced obesity
Source: Front Endocrinol (Lausanne). 2023 Jan 9;13:983827. doi: 10.3389/fendo.2022.983827 (PMC9869264; doi:10.3389/fendo.2022.983827)

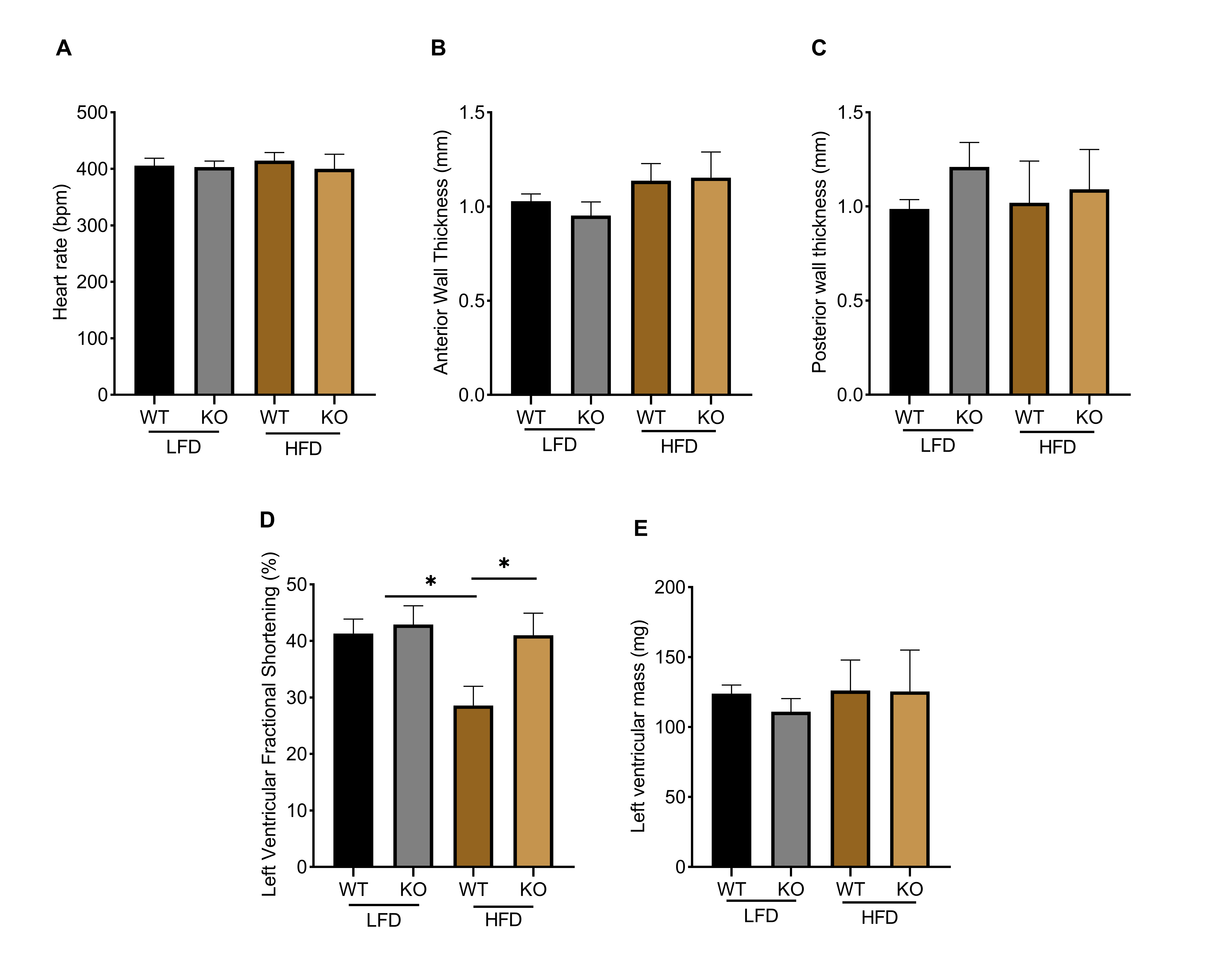

Supplement: Supplementary file 1 [file Image_1.tif]

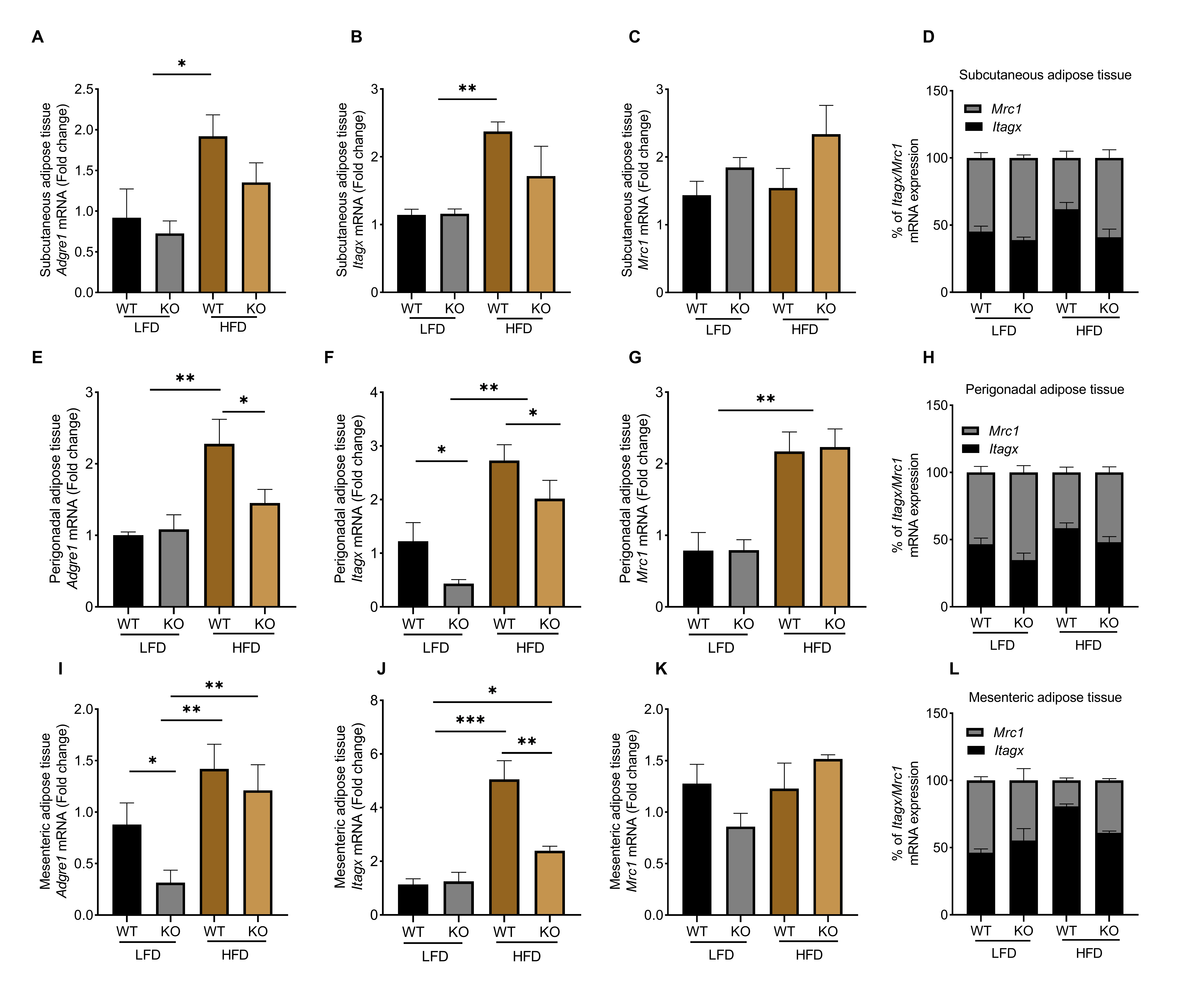

Supplement: Supplementary file 2 [file Image_2.tif]
