# Supplementary material for: Triggering receptor expressed on myeloid cells-1 deletion in mice attenuates high-fat diet-induced obesity
Source: Front Endocrinol (Lausanne). 2023 Jan 9;13:983827. doi: 10.3389/fendo.2022.983827 (PMC9869264; doi:10.3389/fendo.2022.983827)
Supplement: Supplementary file 3 [file DataSheet_1.docx]

Supplementary Material

# Supplementary material

Real-Time PCR

Frozen liver, subcutaneous, mesenteric, and perigonadal white adipose tissue were cut into small pieces, kept frozen, and ground in a homogenizer (FastPrep-24, MP biomedicals SARL, France) using ceramic balls 1.4 mm in diameter in the presence of Trizol reagent (Thermo Fisher Scientific, USA) to extract total RNA. RNA purification was performed using RNAeasy Plus Mini Kit (Qiagen, Netherlands) according to the manufacturer's instructions and quantified with MicroDrop (Thermo Fisher Scientific, USA) to determine RNA purity and concentration. Samples were diluted to the same concentration before being reverse transcribed using the iScript cDNA synthesis kit (Bio-Rad, USA) and quantified by quantitative polymerase chain reaction (PCR) using Qiagen probes (Quantitect Primers) for murine *Adgre-1, Itagx,* and *Mrc-1* . Murine *Gapdh*, *β-actin*, *Rna18S* probes served as housekeeping genes.

Echocardiography

Transthoracic echocardiography was performed 12 weeks after the start of the diet using a VisualSonics 3100 system. Left Ventricular Ejection Fraction and other indices of systolic function were obtained from parasternal long axis B-mode scans. Anesthesia was induced by 5% isoflurane with 1.5 L/min of O_2_ and confirmed by a lack of response to firm pressure on one of the hind paws. During echocardiography acquisition, isoflurane and O_2_ were reduced to 2 % and 0.5 L/min respectively. The body-temperature was monitored during the procedure. Anterior Wall Thickness (AWT), Left Ventricular Systolic Dimension (LVSD), Left Ventricular Diastolic Dimension (LVDD), and Posterior Wall Thickness (PWT) were measured, and Left Ventricular Fractional Shortening (LVFS) and Left Ventricular Mass (LVM) were calculated.

# Supplementary Figures

**Supplementary Figure 1:** Quantification of *Adgre1*, *Itagx* and *Mrc1* mRNA expression determined by real-time qPCR in **(A-D)** subcutaneous, **(E-H)** perigonadal, and **(I-L)** mesenteric adipose tissue from wild-Type (WT) or TREM-1 Knock-Out (KO) fed with control Low Fat Diet (LFD) or High Fat Diet (HFD). *Itagx/Mrc1* ratio calculated from qPCR ratio. Data are presented as mean ± SEM, n=5 mice per group (Kruskal Wallis test *p < 0.05, **p < 0.01, ***p < 0.001).

**Supplementary Figure 2:** Echocardiography was performed at 12 weeks on Wild-Type (WT) or *Trem*-1 Knock-Out (KO) fed with control Low Fat Diet (LFD) or High Fat Diet (HFD). **(A)** Heart rate, **(B)** anterior wall thickness, **(C)** posterior wall thickness, **(D)** left ventricular fractional shortening, and **(E)** Left ventricular mass. Data are presented as mean ± SEM n=6-10 mice per group (One-way ANOVA analysis *p < 0.05).
